# Outdoor residual spraying for malaria vector-control in Kayin (Karen) state, Myanmar: A cluster randomized controlled trial

**DOI:** 10.1371/journal.pone.0274320

**Published:** 2022-09-09

**Authors:** Victor Chaumeau, Ladda Kajeechiwa, Thithiworada Kulabkeeree, Sunisa Sawasdichai, Warat Haohankhunnatham, Aritsara Inta, Monthicha Phanaphadungtham, Florian Girond, Vincent Herbreteau, Gilles Delmas, François Nosten

**Affiliations:** 1 Shoklo Malaria Research Unit, Mahidol-Oxford Research Unit, Faculty of Tropical Medicine, Mahidol University, Mae Sot, Thailand; 2 Centre for Tropical Medicine and Global Health, Nuffield Department of Medicine, University of Oxford, Oxford, United Kingdom; 3 Institut Pasteur du Cambodge, Institut Pasteur International Network, Phnom Penh, Cambodia; 4 Institut de Recherche pour le Développement, UMR 228 Espace-Dev (IRD, UA, UG, UM, UR), Phnom Penh, Cambodia; Department of Medical Research (Lower Myanmar) Advanced Molecular Research Center, MYANMAR

## Abstract

Outdoor and early biting by mosquitoes challenge the efficacy of bed nets and indoor residual spraying against malaria in the Greater Mekong Subregion. The objective of this study was to assess the efficacy of outdoor residual spraying (ORS) for malaria vector-control in this region. A cluster randomized controlled trial was conducted between July 2018 and April 2019 in twelve villages in Karen (Kayin) state, Myanmar. Villages were randomly assigned to receive either a single round of ORS with a capsule suspension of lambda-cyhalothrin for two days in October or no intervention (six villages per group). The primary endpoint was the biting rate of malaria mosquitoes assessed with human-landing catch and cow-baited trap collection methods, and was analyzed with a Bayesian multi-level model. In the intervention villages, the proportion of households located within the sprayed area ranged between 42 and 100% and the application rate ranged between 63 and 559 g of active ingredient per hectare. At baseline, the median of *Anopheles* biting rate estimates in the twelve villages was 2 bites per person per night (inter-quartile range [IQR] 0–5, range 0–48) indoors, 6 bites per person per night (IQR 2–16, range 0–342) outdoors and 206 bites per cow per night (IQR 83–380, range 19–1149) in the cow-baited trap. In intention-to-treat analysis, it was estimated that ORS reduced biting rate by 72% (95% confidence interval [CI] 63–79) from Month 0 to Month 3 and by 79% (95% CI 62–88) from Month 4 to Month 6, considering control villages as the reference. In conclusion, ORS rapidly reduces the biting rates of malaria mosquitoes in a Southeast Asian setting where the vectors bite mostly outdoors and at a time when people are not protected by mosquito bed nets.

## Introduction

Kayin (Karen) state is located in Eastern Myanmar and borders Thailand. An estimated 450,000 people living in these rural areas are exposed to malaria. Transmission is low, seasonal and unstable and multi-drug resistant falciparum malaria is a major concern [1]. An aggressive elimination program was started in 2013 with the objective of eliminating *Plasmodium falciparum* as quickly as possible to slow down the spread of resistance to the artemisinin derivatives [2]. Falciparum malaria was eliminated from more than 90% of the villages with widespread deployment of early diagnosis and treatment, and targeted mass drug administration campaigns in places where the prevalence of submicroscopic infection was high [3]. Some foci of highly resistant parasites have persisted in the North of Kayin state where ecological, social and political factors have posed additional challenges to elimination. *P*. *vivax* is now the main cause of malaria in this population. It displays the short latency frequently relapsing phenotype [4, 5] and the majority of clinical cases are caused by relapsing hypnozoites [6]. Although the endemicity of vivax malaria has also declined in recent years [7], it is more difficult to tackle than falciparum malaria because of the dormant liver stages, high transmission potential during the early phase of infection [8], short extrinsic incubation period and low thermal limit for schizogony in the mosquito [9].

In this region, the primary vectors are *Anopheles minimus* (Minimus Complex, Funestus Group), *An*. *maculatus*, *An*. *sawadwongporni* (Maculatus Group), *An*. *dirus* and *An*. *baimaii* (Dirus Complex, Leucosphyrus Group). *Anopheles pseudowillmori* (Maculatus Group), *An*. *aconitus* (Aconitus Subgroup, Funestus Group) and some members in the Annularis and Barbirostris Groups are secondary vectors [10]. Transmission occurs throughout the year with two seasonal peaks in May-July and December-January [1, 10]. Biting rates can be very high, thereby playing a disproportionate role in driving transmission intensity in this setting where the prevalence of *Plasmodium* infection in mosquito populations is low [11]. Bed nets and indoor residual spraying fail to prevent most malaria infections [12, 13] because of the ecology and biology of relevant *Anopheles* species, including exophily and exophagy, zoophagy and opportunistic blood type selection, and activity peaks at dusk and dawn [10, 14, 15]. Larval source management is difficult to implement because of the diverse and fragmented nature of larval habitats [16], and because incredibly high densities of vector larvae can be found over large areas covered with paddy fields [17]. Several vector species multiply in a variety of biotopes and at different times of the year, adding another layer of complexity to the dynamics of entomological indices.

In order to avoid severe desiccation and heat stress during daytime, mosquitoes seek out resting habitats that provide a fresh and humid microclimate [18]. Daytime resting habitats have been identified both indoors (*e*.*g*. roof, wall, ceilings of houses, animal barns) and outdoors (*e*.*g*. tree holes, rodent holes, dense bushes, wells) [19]. We hypothesized that peridomestic dense bushes in and around the village are the main daytime resting habitat of *Anopheles* mosquitoes in Kayin state, and therefore proposed focused outdoor residual spraying (ORS) for malaria vector-control in this region. Several published studies have assessed the duration and magnitude of the insecticidal effects of insecticide mists applied to outdoor vegetation (reviewed in [20]). Only one study used malaria mosquitoes, relevant exposure time and modern formulation of insecticides: in a standard forced-contact assay with laboratory-adapted colony of pyrethroid-susceptible *An*. *dirus*, the residual effects of a capsule suspension of lambda-cyhalothrin sprayed on outdoor vegetation at a target concentration of 500 g of active ingredient per hectare lasted for several weeks to months during the rainy and dry seasons respectively [20]. Furthermore, the effects of ORS on wild populations of malaria mosquitoes are not well documented (reviewed in [21]). In a pilot study in four villages, ORS with a capsule suspension of lambda-cyhalothrin sprayed rapidly reduced the biting rate of malaria vectors but intervention impact on additional outcome measures (e.g. vector longevity and resistance, malaria incidence) was not assessed [21]. The objective of the herein study was to further assess at scale the efficacy of ORS for malaria vector-control in Kayin state, thereby contributing to knowledge of the impact of vector-control on the entomological indices, vector resistance and vivax malaria in this region.

## Materials and methods

### Study design

A randomized controlled trial was conducted in 12 villages selected among the 1,200 malaria posts operated by the Malaria Elimination Task Force in Kayin state on the willingness of villagers to participate in the study and accessibility of the sites. The villages were randomly assigned to receive either a single round of ORS with lambda-cyhalothrin in October 2018 or no intervention by block randomization (six villages per group). Entomological surveys were conducted monthly in all villages for three months before (baseline) and six months after the intervention (Month 1 to Month 6). Entomological surveys were repeated immediately after the intervention (at Month 0) only in the sprayed villages to assess the immediate impact of ORS on mosquitoes. Collected specimens were used to estimate the biting rates, the proportion of *Plasmodium*-infected vectors, of parous females, and of insecticide resistant phenotypes in mosquito populations. A population census of the villagers was organized at the beginning of the study. All symptomatic malaria cases detected by passive surveillance at the village malaria post between November 1^st^, 2017 and November 1^st^, 2019 were recorded and included in the analysis. This time window was chosen since the prevention of new *P*. *vivax* infections with vector control is expected to have a lagged impact on incidence because of the relapses. Normalized Difference Vegetation Index (NDVI), rainfall, air temperature and dew point were used as covariates in the analysis of biting rates in order to consider differences in the environment of the twelve villages and seasonality.

### Randomization and masking

The intervention was allocated to the villages by a block randomization based on baseline entomological data (S1 Table). Villages were ranked for the proportion of *Anopheles* among the total number of collected mosquitoes, the human-biting rate of *Anopheles* mosquitoes, and the ratio between the human-biting and cow-biting rates of *Anopheles* mosquitoes; the ranks were multiplied by 10, 3 and 1 respectively. The ranking variables and weight values were chosen arbitrarily such as to constitute two groups of villages with similar malaria transmission indices. The three weighted ranks were summed and villages were sorted in six blocks of two villages having successive ranks. In each block, villages were randomly assigned to the ORS or control group using computer-generated random numbers. Randomization was performed by a statistician not in the study team using anonymized village codes, without allocation concealment. Only the laboratory personnel who processed the samples was masked to group assignment.

### Procedures

A subset of 40 villages located near the border was selected among the list of malaria posts. Villages difficult to access were removed from the list. Then, a meeting was organized between the study team, local authorities and headmen of the remaining villages to constitute the final list of 12 villages. The twelve villages were selected by mean of mutual agreement between community members and the study team had no role in the final selection process. ORS was carried out in six villages between October 2^nd^ and October 17^th^ for two days per village as previously described [21]. No intervention was carried out in the villages of the control group. Entomological surveys were conducted as previously described [21] and yielded 25 person-nights of collection indoors, 25 person-nights outdoors and 5 cow-nights per survey. *Anopheles* specimens were sorted macroscopically and then identified using a dichotomic identification key [22]. Malaria mosquitoes captured in the cow-baited trap and still alive at the end of the survey were used to perform standard insecticide susceptibility tests with papers impregnated with 18 mg/m^2^ of lambda-cyhalothrin as previously described [21]. Mosquito susceptibility to additional insecticides, including deltamethrin (18 mg of active ingredient [a.i.] /m2), permethrin (275 mg of a.i. /m2), bendiocarb (40 mg of a.i. /m2), propoxur (40 mg of a.i. /m2) or D.D.T. (1787 mg of a.i. /m2), was also assessed at baseline when at least 50 specimens of a given species in a given village were tested with lambda-cyhalothrin. The remaining specimens were kept at -20˚C in 1.5 ml plastic tubes containing silica gel until further processing. Parity rate was assessed in the specimens of the Funestus, Maculatus and Leucosphyrus groups collected by human-landing catches using the Detinova method [23]. The abdomen of dry-frozen mosquito specimens was separated from the cephalothorax and rehydrated overnight in 0.1% sodium dodecyl-sulfate solution at ambient temperature. Ovaries were dissected from the rehydrated abdomen into 1X phosphate buffer saline and dried on a glass slide overnight. Slides were examined under a microscope. Specimens having coiled tracheations were classified as nulliparous and those with uncoiled tracheation were classified as parous. All slides were read twice by blinded operators and discrepancies were resolved by a third operator. The cephalothorax was screened for *Plasmodium* sporozoites with a PCR assay previously described using the primer pair PL1473F18/PL1679R18 [24]. NDVI was calculated using Sentinel-2 satellite images acquired during the study period. Daily rainfall data, mean air temperature at 2m and dewpoint temperature at 2m were collected using a combination of R and Google Earth Engine with the *rgee* R package [25].

### Outcomes

The primary endpoint was the biting rate of malaria mosquitoes (defined as the number of collected *Anopheles* per man per night). The secondary outcomes were the proportion of lambda-cyhalothrin resistant mosquitoes (defined as the number of specimens alive at the end of the test divided by the total number of exposed specimens), the proportion of parous female mosquitoes (defined as the number of specimens having uncoiled ovarian tracheations divided by the total number mosquitoes with either uncoiled or coiled tracheations) in the mosquito populations and the incidence rate of malaria (defined as the number of passively detected symptomatic cases reported by the village malaria post per 1000 persons per month).

### Data analysis

In descriptive analyses, Poisson and binomial 95% confidence interval (CI) were calculated for count data and proportions respectively, using exact methods. The impact of ORS on the outcomes was estimated using Bayesian multi-level models. Mosquito-biting rates were modelled using a negative binomial family function and a varying intercept for each collection site. The unit of the model was the person-night of collection. Mean of daily air temperature, dew point, NDVI (averaged over the month preceding the collection night), cumulative rainfall over the month preceding the collection night, intervention group (control or ORS), follow-up period (baseline, Month 0 to Month 3 and Month 4 to Month 6) and collection method (indoor or outdoor human-landing catch, and cow-baited trap) were included as constant effect predictors. Continuous variables were transformed into categorical variables using quartile values to relax the assumption of a linear relationship with the outcome. An interaction term between the intervention group, follow-up period and collection method was introduced, allowing comparisons of mosquito biting rates (indoors, outdoors and in the cow-baited trap) between the two intervention groups at different time points. A binomial family function was used for the analysis of the proportions of lambda-cyhalothrin phenotypes and parous females in mosquito populations. The unit in the models was the proportion of parous or resistant specimens collated by species, village and survey. Mosquito taxon, season, intervention group, follow-up period and an interaction term between the intervention group and follow-up period were introduced as constant effect predictors, and a varying intercept for each village was specified. A Poisson family function was used for the model of malaria incidence. The unit of the model was the daily number of clinical cases reported by the village malaria post. The season, age group (0 to 15 years, and more than 15 years), intervention group, follow-up period and an interaction term between the intervention group and follow-up period were introduced as constant effect predictors, and a varying intercept for each village was specified. The population size (natural log-transformed) was included as an offset. ORS impact on outcomes was estimated using the coefficient of the interaction term between the intervention group and follow-up period, considering control villages as the reference. All models were created in Stan computational framework [26] accessed with *brms* R package [27]. To improve convergence and guard against overfitting, mildly informative conservative priors were specified. The analyses were performed according to the intention-to-treat principle including all observations in the final dataset.

### Ethics

The study was approved by the Oxford Tropical Research Ethics Committee (reference 17–18), the Karen Department of Health and Welfare, Karen National Union and the Tak Province Border Community Ethics Advisory Board [28]. All participants provided their written informed consent to participate in the study. The land accessed is protected by the local Karen authorities. No sensitive animals or plants were sampled.

## Results

**Fig 1:**
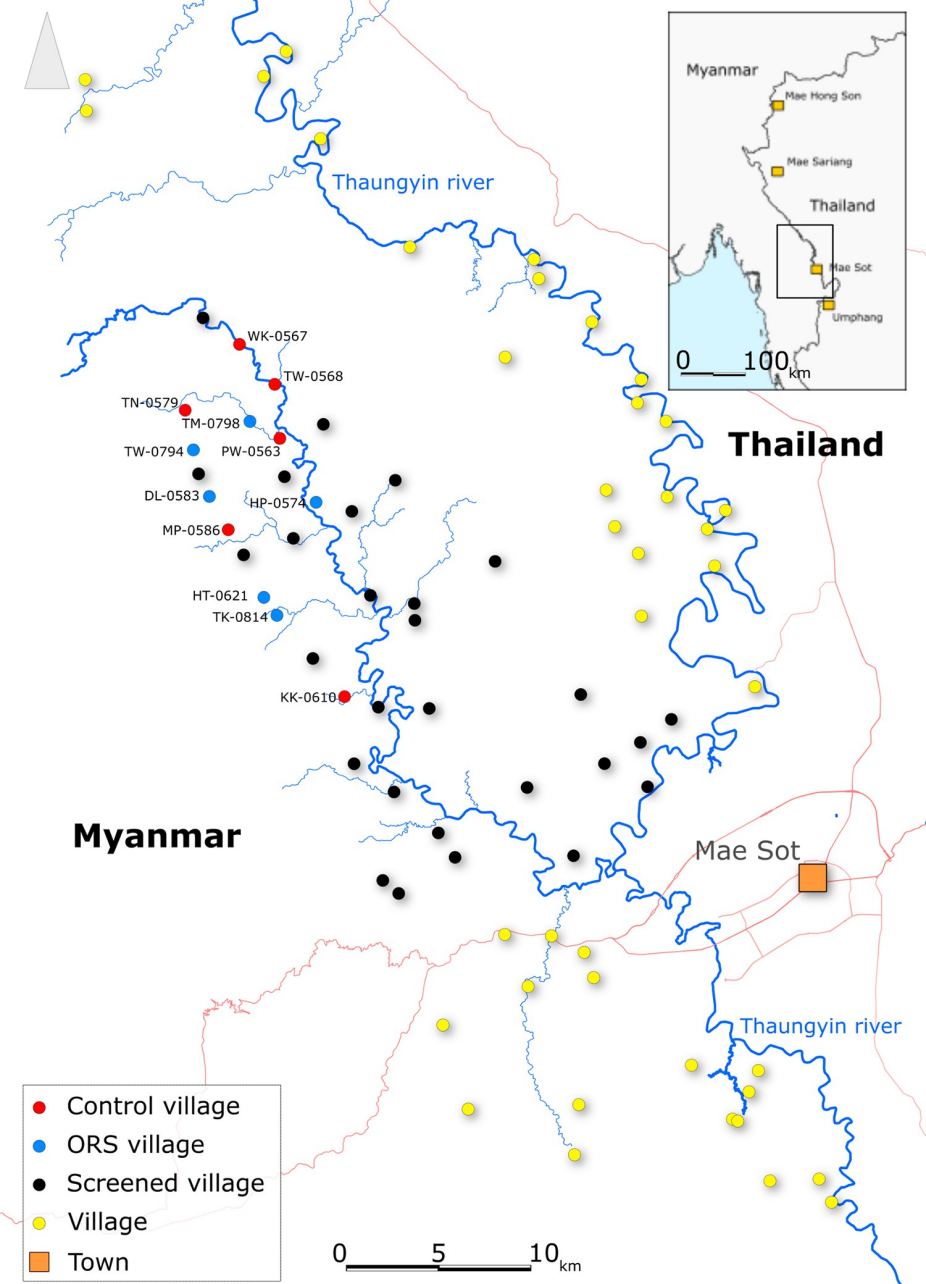
Map of the study area. Contains information from OpenStreetMap and OpenStreetMap Foundation, which is made available under the Open Database License.

Out of the 40 villages screened, 12 were selected and assigned either to the ORS or no intervention group (Fig 1). The villages were groupings of households surrounded by forest and paddy fields, of which some were divided into two or more clusters of households located 50 to 300 meters apart one from another. Population size was approximately 20% smaller in the ORS group than in the control (Table 1). 40% of the population was less than 15 years old; the main occupations of people more than 15 years old were rice farming, homeworking and education. Heavy rainfall caused by particularly intense monsoon rains on that year resulted in flooding of the study sites for approximately three weeks in July 2018.

**Table 1:** Demographic and environmental characteristics of the villages in the two groups.

	Median	Range	Overall
Number of inhabitants per village			
Control	360	284–858	2958
ORS	295	220–773	2378
Number of households per village			
Control	59	50–181	573
ORS	60	45–160	474
Number of people per household			
Control	5	1–15	5.2
ORS	5	1–17	5
Median age (in years)			
Control	20	0.1–109	25
ORS	20	0.1–99	24.1
Sex ratio (male/female)			
Control	1.01	0.91–1.07	0.99
ORS	1.03	0.96–1.22	1.02
Area (ha)			
Control	26	9–50	157
ORS	15	7–48	133

Table 2 shows the characteristics of the ORS intervention. Only one village received the target application rate and full coverage. Some villagers of the first sprayed village refused the intervention, hence the low application rate. The main concern of the villagers was the potential toxicity of ORS to domestic animals, which constitute an important source of food and income for the household. Only half of the second and third villages was sprayed, and only half of the target application rate was achieved in the fifth and sixth villages. In these villages, the surface area was too large or access to the sites was too difficult to complete the intervention in two days.

**Table 2:** Summary of the characteristics of ORS intervention by village.

	TK-0814	HT-0621	DL-0583	TW-0794	HP-0574	TM-0798
Date of intervention	Oct. 2–3	Oct. 5–6	Oct. 8–9	Oct. 11–12	Oct. 14–15	Oct. 17–18
Outer perimeter (km)	2.0	1.4	0.9	1.1	1.8	1.1
Inner area (ha)	31	11	5	8	18	8
Area coverage (%)	65	32	41	99	99	70
Household coverage (%)	81	49	42	100	98	72
Population coverage (%)	83	47	44	100	98	70
Total amount of a.i. (g)	1,950	3,750	2,875	3,950	4,575	4,225
Application rate (g a.i. /ha)	63	339	559	516	248	526

a.i., active ingredient.

**Fig 2:**
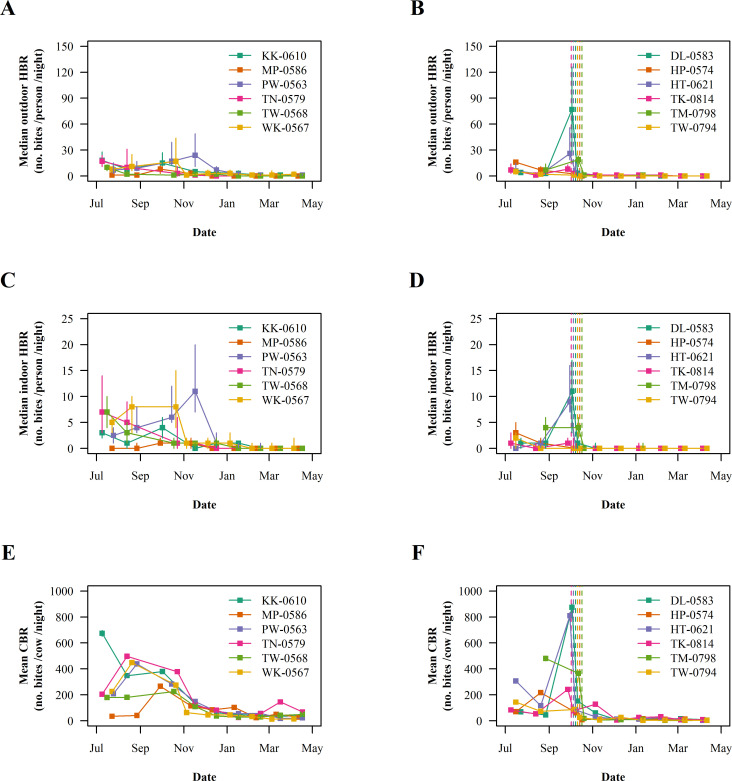
Evolution of the median biting rates of malaria mosquitoes during the follow-up. (A) Indoor human-biting rate in the control villages, (B) indoor human-biting rate in the sprayed villages, (C) outdoor human-biting rate in the control villages, (D) outdoor human-biting rate in the sprayed villages, (E) cow-biting rate in the control villages and (F) cow-biting rate in the sprayed villages. Vertical solid bars show the interquartile range. Vertical dashed lines show intervention dates.

**Fig 3:**
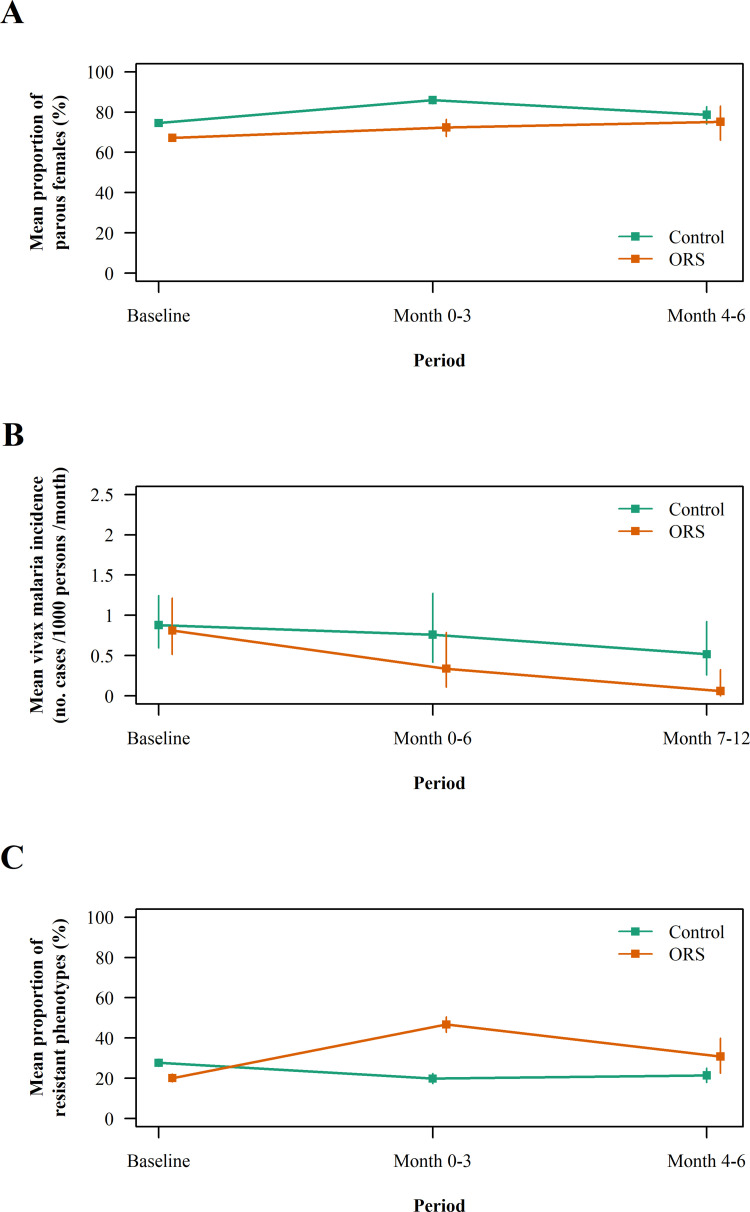
Evolution of the secondary outcomes in the village of the ORS and no intervention groups. (A) Mean proportion of parous *Anopheles* females, (B) mean incidence of vivax malaria and (C) mean proportion of lambda-cyhalothrin resistant phenotypes in *Anopheles* of the control and sprayed villages during the study. Vertical bars show the 95% confidence intervals.

Baseline characteristics of the entomological indices in the control and sprayed villages are summarized in Table 3. The diversity of malaria mosquitoes was high and the most abundant human-biting species were *An*. *maculatus* (37%), *An*. *minimus* (36%) and *An*. *annularis* (12%). Malaria mosquitoes were strongly exophagic and zoophagic; the proportion of outdoor and early biters was very high. Biting rates were significantly lower in the ORS group than in the control (IRR 0.55, 95% CI 0.37–0.82). The median of observed biting rate estimates in the villages of the control group was 3 bites per person per night (inter-quartile range [IQR] 1–6, range 0–48) indoors, 8 bites per person per night (IQR 3–17, range 0–110) outdoors and 282 bites per cow per night (IQR 175–412, range 19–798) in the cow-baited trap. The corresponding figures in the sprayed villages was 1 bite per person per night (IQR 0–4, range 0–42), 5 bites per person per night (IQR 2–13, range 0–342) and 112 bites per cow per night (IQR 63–343, range 23–1149). Immediately after ORS, the median of observed biting rate estimates in the sprayed villages dropped to 0 bites per person per night (IQR 0–1, range 0–19) indoors, 2 bites per person per night (IQR 0–4, range 0–31) outdoors and 44 bites per cow per night (IQR 12–86, range 0–221) in the cow-baited trap (Fig 2). In intention-to-treat analysis, it was estimated that ORS reduced the biting rate by 70 to 80% between Month 0 and Month 6 (Fig 2 and Table 4); ORS impact on biting rate was similar indoors, outdoors and in the cow-baited trap. At baseline, the observed proportion of parous female mosquitoes ranged between 62% and 79% by village and was higher in *An*. *minimus* (83%, 95% CI = 81–84) than in *An*. *maculatus* (62%, 95% CI = 60–63) and *An*. *dirus* (68%, 95% CI = 59–76). ORS was associated with a one-third reduction of parity rate between Month 0 and Month 3 considering control group as the reference (Fig 3A and Table 4). The overall *P*. *vivax*-sporozoite index at baseline was 0.022% (95% CI 0.003–0.078). The resulting *P*. *vivax* entomological inoculation rate was 0.0014 infective bites/person/night (95% CI 0.0002–0.0052), precluding further assessment of the impact of ORS on the entomological indices of transmission.

**Table 3:** Baseline characteristics of the entomological indices in the villages of the ORS and no intervention (control) groups.

	Median	Range	Overall
Proportion of *Anopheles* mosquitoes (%)			
Control	55	35–82	58
ORS	67	23–84	61
Mean outdoor human-biting rate/mean indoor human-biting rate			
Control	2.7	1.4–6.0	2.7
ORS	4.3	3.0–6.9	4.7
Mean cow-biting rate/mean indoor human-biting rate			
Control	54	36–154	60
ORS	84	59–115	80
Mean proportion of outdoor and early mosquito biters (%)			
Control	90	85–95	90
ORS	93	89–98	93
Mean proportion of parous females (%)			
Control	74	63–79	75
ORS	69	62–79	67
Mean human-biting rate (number of bites/person/night)			
Control	7.6	2.0–9.7	6.7
ORS	5.5	2.2–16	6.7
Mean *P*. *vivax* sporozoite index (%)			
Control	0	0–0.082	0.033
ORS	0	0–0	0
Mean *P*. *vivax*-entomological inoculation rate (number of infective bites/person/night)			
Control	0	0–0.007	0.002
ORS	0	0–0	0
Mean proportion of lambda-cyhalothrin resistant phenotypes (%)			
Control	71	66–83	72
ORS	80	68–95	80

**Table 4:** ORS impact on the study outcomes during the follow-up.

	IRR or OR^a^	95% CI
Biting rate		
Month 0 to Month 3	0.28	0.21–0.37
Month 4 to Month 6	0.21	0.12–0.38
Proportion of parous females		
Month 0 to Month 3	0.65	0.48–0.87
Month 4 to Month 6	1.15	0.67–1.98
Vivax malaria incidence		
Month 0 to Month 6	0.46	0.13–1.43
Month 7 to Month 12	0.09	0.01–0.63
Proportion of lambda-cyhalothrin resistant phenotypes		
Month 0 to Month 3	2.70	2.00–3.65
Month 4 to Month 6	3.58	2.11–6.03

CI, confidence interval; IRR, incidence rate ration, OR, odds ratio.

^a^ Coefficient estimate for the interaction term between the ORS group and the follow-up period, considering the control as the reference.

At baseline, the observed mean incidence rate of vivax malaria was 0.88 cases per 1000 persons per month (95% CI 0.60–1.25) and 0.81 cases per 1000 persons per month (95% CI 0.51–1.22) in the control and ORS group respectively. The burden was higher in children and adolescents than in adults (IRR 2.76, 95% CI 1.76–4.38) and during the rainy season than the dry season (IRR 1.86, 95% CI 1.15–3.03). After ORS, vivax malaria incidence decreased more in the sprayed villages than in the control. Between Month 0 and Month 6, the incidence rate was 0.82 cases per 1000 persons per month (95% CI 0.45–1.38) versus 0.36 cases per 1000 persons per month (95% CI 0.12–0.86) in the control and ORS group respectively; the corresponding figure between Month 7 and Month 12 was 0.54 cases per 1000 persons per month (95% CI 0.27–0.96) and 0.06 cases per 1000 persons per month (95% CI 0.001–0.33) (Fig 3B and Table 4). Differences between the two groups were not statistically robust and confidence intervals were broad because the number of cases and clusters were small. Consistent with the elimination of *P*. *falciparum* in the study area, no falciparum malaria case or infected mosquito was detected.

During baseline surveys, the observed proportion of lambda-cyhalothrin resistant phenotypes ranged between 4% (95% CI 0.4–13) and 73% (95% CI 66–80) in *An*. *kochi* and *An*. *hyrcanus* respectively (S2 Table), and was significantly lower in the ORS group than in the control (IRR 0., 95% CI 0.38–1.04). Higher prevalence of resistant phenotypes was associated with a decreased proportion of knocked-down specimens in five taxa. Phenotypes resistant to deltamethrin, permethrin, bendiocarb and/or propoxur were less frequently detected. After ORS, the proportion of lambda-cyhalothrin resistant phenotypes increased in the sprayed villages but not in the control (Fig 3C and Table 4).

## Discussion

The efficacy of ORS for malaria vector control was evaluated in Kayin state where transmission displays typical features of the Southeast Asian setting. The target application rate and full coverage were achieved in only one out of six villages, highlighting the challenges faced to engage the communities and logistic constraints that apply to this intervention (i.e. supply transportation, spraying duration and site accessibility) [29]. Nevertheless, a single round of ORS during the rainy season decreased the biting rate and longevity of multiple vector species, and the incidence of *P*. *vivax*-infection in an area where 20–30% of the malaria mosquitoes expressed a phenotype conferring resistance to the sprayed insecticide at baseline. These results confirm the hypothesis that application of residual insecticides to the peridomestic dense vegetation inside and around the village can rapidly reduce transmission and prevent new infections in an area where outdoor and early exposure to mosquito bites challenge the efficacy of mosquito bed nets and indoor residual spraying [20, 21].

ORS selected for lambda-cyhalothrin resistant phenotypes in mosquito populations. Similar observation was made after operational deployment of insecticide-treated bed nets and indoor residual spraying in Africa [30–33]. Slow restoration of susceptibility was observed after removal of the insecticide in *An*. *gambiae* selected for resistance in the laboratory [34] but the dynamics of resistances in natural settings remain unknown. Furthermore, the interactions between vector resistance, malaria transmission and the outcome of vector-control are complex and not well understood [35]. For example, there was no association between the prevalence of resistant phenotypes in mosquito populations and the efficacy of insecticide-treated bed-nets in a large multi-centre study conducted in some African countries and India [36] but addition of piperonyl-butoxide or a second insecticide to pyrethroid-treated bed nets provided additional levels of protection against malaria infection in Tanzania and Uganda [37–39]. Repeated exposure and delayed mortality were identified as major factors contributing to the cut in transmission potential of resistant vectors [40]. Lack of delayed mortality has been reported recently in some natural populations of mosquitoes with high levels of resistance in Burkina Faso and Cameron [41, 42] but the consequences on the efficacy of vector-control interventions have not been assessed.

The impact of ORS on the entomological indices resulted in a gradual reduction of vivax malaria incidence for one year after the intervention yet the confidence intervals around coefficient estimates were broad because the numbers of cases and clusters were small. The impact of vector-control on vivax malaria is poorly understood because relapses confound the relationship between the incidence of clinical cases and the entomological inoculation rate, and because existing interventions have only a limited efficacy against the vectors of *P*. *vivax* [43]. It is generally accepted that “vector-control has no impact on the human reservoir of latent hypnozoites” [44]. By preventing reinfections, vector-control is expected to reduce not only the rate of primary attacks but also the number of hypnozoites in the liver of individuals exposed to malaria mosquito bites when compared to individuals protected by the intervention (and hence the rate of subsequent relapses). Moreover, in the absence of reinfection, the number of hypnozoites decreases over time as relapses occur. Therefore, if effective interruption of transmission is achieved, the reservoir of hypnozoites is expected to drain spontaneously after a certain yet unknown period of time. Since one inoculation can give several relapses, it is likely that prevention of new infections would result in an important reduction of the incidence of clinical cases (with a lag corresponding to the timing of relapses).

This study had several limitations. Although baseline differences in overall biting rate between the two intervention groups were considered in the model, heterogenous species-specific dynamics across sites could have biased our estimation of ORS impact on biting rate. Deciphering the complex interactions between mosquito populations and the environment would have been relevant to the study but it would have required a different modelling framework and to identify several thousands of mosquitoes with molecular methods, which was not possible to implement. Nevertheless, ORS impact on biting rate is supported by the concomitant observations on parity, resistance and malaria incidence. Mosquito biting rates do not integrate man behaviors and personal protection provided by concomitant vector control measures. Therefore, it does not reflect actual exposure accurately. Dry blood spot specimens were collected during cross-sectional surveys conducted during baseline and after the intervention to assess antibody responses to *Anopheles* salivary antigens, a promising approach to measure changes in transmission dynamics and evaluate the efficacy of vector control in this setting [45, 46]. The impact of ORS on the antibody responses to salivary antigens is ongoing and will be reported separately. Previous work showed marked differences in the duration of the residual effects of insecticide mist during the rainy and dry seasons [20], but the specific effects of rains on intervention outcomes were not assessed in this study. Addressing this question would have required a different design and may be the focus of future studies. Submicroscopic reservoirs of malaria parasites were not assessed and therefore could not assess intervention impact on the circulating forms of the *P*. *vivax*. The effects of ORS on the levels and mechanisms of vector resistances was not assessed. The feasibility of doing additional analyses was limited by the numbers of collected specimens still alive at the end of the surveys and by the absence of molecular makers of insecticide resistance in these mosquito species. Data on non-target organisms were not collected precluding assessment of the impact of ORS on the environment. The adverse effects of ORS are likely to be important at the sprayed sites and warrant careful consideration. A detailed risk assessment was published previously [21]. The environmental fate of the sprayed insecticide, size of the ecosystem and location of the habitat of endangered species lower the risks of ORS having a major impact on the environment. The sprayed villages were monitored closely after the intervention and there were no complaints from the villagers about ORS impact on their environment.

The impact of ORS on malaria is explained by its effects on the entomological indices of transmission and more precisely on transmission intensity and vectorial capacity. At first, ORS rapidly reduces the abundance and biting rate of the adult mosquito population probably through mass killing effect (upon contact of resting mosquitoes with the insecticide mist) and deterrence. Then, the residual effects probably continue to kill, deter and most importantly to reduce the longevity of the next generations of mosquitoes for several weeks [20, 21]. The reduction of vector longevity (as was assessed with the parity rate) is likely an important factor and contributes to both the decrease in vector abundance (by reducing the number of gonotrophic cycles and hence of the size of the progeny) and in the transmission potential of infected vectors [47]. Both male and female *Anopheles* mosquitoes rest on outdoor vegetation and intervention impact on male mosquitoes is also probably important. Altogether, the effects of ORS on vector abundance and longevity reduces transmission intensity, vectorial capacity and subsequently, malaria incidence.

The impact of ORS on mosquito biting rates was large despite application rates lower than the target, suggesting that smaller quantities of insecticide should be used. Additional studies striving to optimize the protocol for ORS would be valuable to reduce the use of insecticide and maximize intervention efficacy. Moreover, future research should be carried out to characterize the effects of insecticide resistances on transmission and outcome of vector-control interventions. The impact of vector-control on vivax malaria is also an important knowledge gap that warrants being addressed in the context of elimination.

ORS with a capsule suspension of lambda-cyhalothrin rapidly reduces the entomological indices of malaria transmission and prevent new infections in an area of Southeast Asia where the vectors are particularly difficult to control and where pyrethroid resistance has been documented. There are several drawbacks to ORS including the toxicity to the environment, effects of vector resistance and logistic constraints which limit the scale-up of this intervention for malaria vector-control. ORS should be used in combination with other interventions when and where a rapid interruption of transmission is needed, for example during malaria outbreaks, but probably not as a routine vector control measure. Furthermore, this study provides important and rare insights on the impact of vector-control on vivax malaria and highlights the importance of not overlooking prevention of new infections in elimination strategies.

## Supporting information

S1 Table Randomization table used to allocate intervention to the villages enrolled in the study. (DOCX)

S2 Table Insecticide resistance patterns in malaria mosquitoes during baseline surveys. (DOCX)

S1 Dataset Dataset used in the analysis. (XLSX)

## References

[1] Luxemburger C, Thwai KL, White NJ, Webster HK, Kyle DE, Maelankirri L, et al. The epidemiology of malaria in a Karen population on the western border of Thailand. Trans R Soc Trop Med Hyg. 1996;90(2):105–11. Epub 1996/03/01. doi: 10.1016/s0035-9203(96)90102-9. . 8761562

[2] Carrara VI, Lwin KM, Phyo AP, Ashley E, Wiladphaingern J, Sriprawat K, et al. Malaria burden and artemisinin resistance in the mobile and migrant population on the Thai-Myanmar border, 1999–2011: an observational study. PLoS Med. 2013;10(3):e1001398. Epub 2013/03/09. doi: 10.1371/journal.pmed.1001398. ; PubMed Central PMCID: PMC3589269. 23472056 PMC3589269

[3] Landier J, Parker DM, Thu AM, Lwin KM, Delmas G, Nosten FH, et al. Effect of generalised access to early diagnosis and treatment and targeted mass drug administration on *Plasmodium falciparum* malaria in Eastern Myanmar: an observational study of a regional elimination programme. Lancet. 2018;391(10133):1916–26. Epub 2018/04/29. doi: 10.1016/S0140-6736(18)30792-X. ; PubMed Central PMCID: PMC5946089. 29703425 PMC5946089

[4] White NJ. Determinants of relapse periodicity in *Plasmodium vivax* malaria. Malar J. 2011;10:297. Epub 2011/10/13. doi: 10.1186/1475-2875-10-297. ; PubMed Central PMCID: PMC3228849. 21989376 PMC3228849

[5] Battle KE, Karhunen MS, Bhatt S, Gething PW, Howes RE, Golding N, et al. Geographical variation in *Plasmodium vivax* relapse. Malar J. 2014;13:144. Epub 2014/04/16. doi: 10.1186/1475-2875-13-144. ; PubMed Central PMCID: PMC4021508. 24731298 PMC4021508

[6] Taylor AR, Watson JA, Chu CS, Puaprasert K, Duanguppama J, Day NPJ, et al. Resolving the cause of recurrent *Plasmodium vivax* malaria probabilistically. Nat Commun. 2019;10(1):5595. Epub 2019/12/08. doi: 10.1038/s41467-019-13412-x. ; PubMed Central PMCID: PMC6898227. 31811128 PMC6898227

[7] Ashley EA, Phyo AP, Carrara VI, Tun KM, Nosten F, Smithuis F, et al. *Plasmodium vivax* relapse rates following *Plasmodium falciparum* malaria reflect previous transmission intensity. J Infect Dis. 2019;220(1):100–4. Epub 2019/01/31. doi: 10.1093/infdis/jiz052. ; PubMed Central PMCID: PMC6548896. 30698794 PMC6548896

[8] Jeffery GM. The infection of mosquitoes by *Plasmodium vivax* (Chesson strain) during the early primary parasitemias. Am J Trop Med Hyg. 1952;1(4):612–7. Epub 1952/07/01. doi: 10.4269/ajtmh.1952.1.612. . 14943909

[9] Garnham PCC. Malaria parasites and other Haemosporidia: Blackwell Scientific Publications Ltd.; 1966.

[10] Chaumeau V, Fustec B, Nay Hsel S, Montazeau C, Naw Nyo S, Metaane S, et al. Entomological determinants of malaria transmission in Kayin state, Eastern Myanmar: A 24-month longitudinal study in four villages. Wellcome Open Res. 2018;3:109. Epub 2019/06/27. doi: 10.12688/wellcomeopenres.14761.4. ; PubMed Central PMCID: PMC6544137. 31206035 PMC6544137

[11] Chaumeau V, Kajeechiwa L, Fustec B, Landier J, Naw Nyo S, Nay Hsel S, et al. Contribution of asymptomatic *Plasmodium* infections to the transmission of malaria in Kayin state, Myanmar. J Infect Dis. 2019;219(9):1499–509. Epub 2018/12/01. doi: 10.1093/infdis/jiy686. ; PubMed Central PMCID: PMC6467188. 30500927 PMC6467188

[12] Dolan G, ter Kuile FO, Jacoutot V, White NJ, Luxemburger C, Malankirii L, et al. Bed nets for the prevention of malaria and anaemia in pregnancy. Trans R Soc Trop Med Hyg. 1993;87(6):620–6. Epub 1993/11/01. doi: 10.1016/0035-9203(93)90262-o. . 8296357

[13] Smithuis FM, Kyaw MK, Phe UO, van der Broek I, Katterman N, Rogers C, et al. The effect of insecticide-treated bed nets on the incidence and prevalence of malaria in children in an area of unstable seasonal transmission in western Myanmar. Malar J. 2013;12:363. Epub 2013/10/15. doi: 10.1186/1475-2875-12-363. ; PubMed Central PMCID: PMC3854704. 24119916 PMC3854704

[14] Smithuis FM, Kyaw MK, Phe UO, van der Broek I, Katterman N, Rogers C, et al. Entomological determinants of insecticide-treated bed net effectiveness in Western Myanmar. Malar J. 2013;12:364. Epub 2013/10/15. doi: 10.1186/1475-2875-12-364. ; PubMed Central PMCID: PMC4015723. 24119994 PMC4015723

[15] Somboon P, Lines J, Aramrattana A, Chitprarop U, Prajakwong S, Khamboonruang C. Entomological evaluation of community-wide use of lambdacyhalothrin-impregnated bed nets against malaria in a border area of north-west Thailand. Trans R Soc Trop Med Hyg. 1995;89(3):248–54. Epub 1995/05/01. doi: 10.1016/0035-9203(95)90525-1. . 7660424

[16] Rattanarithikul R, Green CA, Panyim S, Noigamol C, Chanaimongkol S, Mahapibul P. Larval habitats of malaria vectors and other *Anopheles* mosquitoes around a transmission focus in northwestern Thailand. J Am Mosq Control Assoc. 1995;11(4):428–33. Epub 1995/12/01. . 8825502

[17] James SP. Extermination of Mosquitos. Ind Med Gaz. 1899;34(11):428. Epub 1899/11/01. ; PubMed Central PMCID: PMC5145717. 29002452 PMC5145717

[18] Dewald JR, Fuller DO, Muller GC, Beier JC. A novel method for mapping village-scale outdoor resting microhabitats of the primary African malaria vector, *Anopheles gambiae*. Malar J. 2016;15(1):489. Epub 2016/09/24. doi: 10.1186/s12936-016-1534-9. ; PubMed Central PMCID: PMC5034649. 27659918 PMC5034649

[19] Silver JB. Mosquito ecology: field sampling methods: Springer Science & Business Media; 2007.

[20] Chaumeau V, Wisisakun P, Sawasdichai S, Kankew P, Htoo GN, Saithanmettajit S, et al. Longevity of the insecticidal effect of three pyrethroid formulations applied to outdoor vegetation on a laboratory-adapted colony of the Southeast Asian malaria vector *Anopheles dirus*. PLoS One. 2020;15(4):e0231251. Epub 2020/04/15. doi: 10.1371/journal.pone.0231251. ; PubMed Central PMCID: PMC7156039. 32287300 PMC7156039

[21] Chaumeau V, Kajeechiwa L, Kulabkeeree T, Vishwakarma RK, Wasisakun P, Hsel SN, et al. Impact of outdoor residual spraying on the biting rate of malaria vectors: A pilot study in four villages in Kayin state, Myanmar. PLoS One. 2020;15(10):e0240598. Epub 2020/10/30. doi: 10.1371/journal.pone.0240598. ; PubMed Central PMCID: PMC7595390. 33119645 PMC7595390

[22] Rattanarithikul R, Harrison BA, Harbach RE, Panthusiri P, Coleman RE, Panthusiri P. Illustrated keys to the mosquitoes of Thailand. IV. *Anopheles*. Southeast Asian J Trop Med Public Health. 2006;37 Suppl 2:1–128. Epub 2007/02/01. . 17262930

[23] Detinova TS. Age-grouping methods in Diptera of medical importance with special reference to some vectors of malaria. Monogr Ser World Health Organ. 1962;47:13–191. Epub 1962/01/01. . 13885800

[24] Chaumeau V, Andolina C, Fustec B, Tuikue Ndam N, Brengues C, Herder S, et al. Comparison of the performances of five primer sets for the detection and quantification of *Plasmodium* in anopheline vectors by real-time PCR. PLoS One. 2016;11(7):e0159160. Epub 2016/07/22. doi: 10.1371/journal.pone.0159160. ; PubMed Central PMCID: PMC4956213. 27441839 PMC4956213

[25] Aybar C, Wu Q, Bautista L, Yali R, Barja A. rgee: An R package for interacting with Google Earth Engine. Journal of Open Source Software. 2020;5(51):2272.

[26] Stan Development Team. Stan Modeling Language Users Guide and Reference Manual, version 2.29 2022. Available from: http://mc-stan.org/.

[27] Bürkner P-C. brms: An R package for Bayesian multilevel models using Stan. Journal of statistical software. 2017;80:1–28.

[28] Cheah PY, Lwin KM, Phaiphun L, Maelankiri L, Parker M, Day NP, et al. Community engagement on the Thai-Burmese border: rationale, experience and lessons learnt. Int Health. 2010;2(2):123–9. Epub 2010/06/01. doi: 10.1016/j.inhe.2010.02.001. ; PubMed Central PMCID: PMC3442337. 22984375 PMC3442337

[29] Tangseefa D, Monthathip K, Tuenpakdee N, Konig A, Kajeechiwa L, Thwin MM, et al. "Nine Dimensions": A multidisciplinary approach for community engagement in a complex postwar border region as part of the targeted malaria elimination in Karen/Kayin State, Myanmar. Wellcome Open Res. 2018;3:116. Epub 2019/03/09. doi: 10.12688/wellcomeopenres.14698.2. ; PubMed Central PMCID: PMC6343222. 30687790 PMC6343222

[30] Vontas J, Grigoraki L, Morgan J, Tsakireli D, Fuseini G, Segura L, et al. Rapid selection of a pyrethroid metabolic enzyme CYP9K1 by operational malaria control activities. Proc Natl Acad Sci U S A. 2018;115(18):4619–24. Epub 2018/04/21. doi: 10.1073/pnas.1719663115. ; PubMed Central PMCID: PMC5939083. 29674455 PMC5939083

[31] Yahouedo GA, Cornelie S, Djegbe I, Ahlonsou J, Aboubakar S, Soares C, et al. Dynamics of pyrethroid resistance in malaria vectors in southern Benin following a large scale implementation of vector control interventions. Parasit Vectors. 2016;9(1):385. Epub 2016/07/06. doi: 10.1186/s13071-016-1661-8. ; PubMed Central PMCID: PMC4932690. 27378358 PMC4932690

[32] Main BJ, Lee Y, Collier TC, Norris LC, Brisco K, Fofana A, et al. Complex genome evolution in *Anopheles coluzzii* associated with increased insecticide usage in Mali. Mol Ecol. 2015;24(20):5145–57. Epub 2015/09/12. doi: 10.1111/mec.13382. ; PubMed Central PMCID: PMC4615556. 26359110 PMC4615556

[33] Implications of Insecticide Resistance Consortium. Implications of insecticide resistance for malaria vector control with long-lasting insecticidal nets: trends in pyrethroid resistance during a WHO-coordinated multi-country prospective study. Parasit Vectors. 2018;11(1):550. Epub 2018/10/24. doi: 10.1186/s13071-018-3101-4. ; PubMed Central PMCID: PMC6198431. 30348209 PMC6198431

[34] Machani MG, Ochomo E, Zhong D, Zhou G, Wang X, Githeko AK, et al. Phenotypic, genotypic and biochemical changes during pyrethroid resistance selection in *Anopheles gambiae* mosquitoes. Sci Rep. 2020;10(1):19063. Epub 2020/11/06. doi: 10.1038/s41598-020-75865-1. ; PubMed Central PMCID: PMC7642378. 33149227 PMC7642378

[35] Thomas MB, Read AF. The threat (or not) of insecticide resistance for malaria control. Proc Natl Acad Sci U S A. 2016;113(32):8900–2. Epub 2016/08/03. doi: 10.1073/pnas.1609889113. ; PubMed Central PMCID: PMC4987779. 27482081 PMC4987779

[36] Kleinschmidt I, Bradley J, Knox TB, Mnzava AP, Kafy HT, Mbogo C, et al. Implications of insecticide resistance for malaria vector control with long-lasting insecticidal nets: a WHO-coordinated, prospective, international, observational cohort study. Lancet Infect Dis. 2018;18(6):640–9. Epub 2018/04/14. doi: 10.1016/S1473-3099(18)30172-5. ; PubMed Central PMCID: PMC5968369. 29650424 PMC5968369

[37] Staedke SG, Gonahasa S, Dorsey G, Kamya MR, Maiteki-Sebuguzi C, Lynd A, et al. Effect of long-lasting insecticidal nets with and without piperonyl butoxide on malaria indicators in Uganda (LLINEUP): a pragmatic, cluster-randomised trial embedded in a national LLIN distribution campaign. Lancet. 2020;395(10232):1292–303. Epub 2020/04/20. doi: 10.1016/S0140-6736(20)30214-2. ; PubMed Central PMCID: PMC7181182. 32305094 PMC7181182

[38] Protopopoff N, Mosha JF, Lukole E, Charlwood JD, Wright A, Mwalimu CD, et al. Effectiveness of a long-lasting piperonyl butoxide-treated insecticidal net and indoor residual spray interventions, separately and together, against malaria transmitted by pyrethroid-resistant mosquitoes: a cluster, randomised controlled, two-by-two factorial design trial. Lancet. 2018;391(10130):1577–88. Epub 2018/04/16. doi: 10.1016/S0140-6736(18)30427-6. ; PubMed Central PMCID: PMC5910376. 29655496 PMC5910376

[39] Mosha JF, Kulkarni MA, Lukole E, Matowo NS, Pitt C, Messenger LA, et al. Effectiveness and cost-effectiveness against malaria of three types of dual-active-ingredient long-lasting insecticidal nets (LLINs) compared with pyrethroid-only LLINs in Tanzania: a four-arm, cluster-randomised trial. Lancet. 2022;399(10331):1227–41. Epub 2022/03/28. doi: 10.1016/S0140-6736(21)02499-5. . 35339225 PMC8971961

[40] Viana M, Hughes A, Matthiopoulos J, Ranson H, Ferguson HM. Delayed mortality effects cut the malaria transmission potential of insecticide-resistant mosquitoes. Proc Natl Acad Sci U S A. 2016;113(32):8975–80. Epub 2016/07/13. doi: 10.1073/pnas.1603431113. ; PubMed Central PMCID: PMC4987804. 27402740 PMC4987804

[41] Hughes A, Lissenden N, Viana M, Toe KH, Ranson H. *Anopheles gambiae* populations from Burkina Faso show minimal delayed mortality after exposure to insecticide-treated nets. Parasit Vectors. 2020;13(1):17. Epub 2020/01/12. doi: 10.1186/s13071-019-3872-2. ; PubMed Central PMCID: PMC6954553. 31924276 PMC6954553

[42] Tchakounte A, Tchouakui M, Mu-Chun C, Tchapga W, Kopia E, Soh PT, et al. Exposure to the insecticide-treated bednet PermaNet 2.0 reduces the longevity of the wild African malaria vector *Anopheles funestus* but GSTe2-resistant mosquitoes live longer. PLoS One. 2019;14(3):e0213949. Epub 2019/03/15. doi: 10.1371/journal.pone.0213949. ; PubMed Central PMCID: PMC6417719. 30870507 PMC6417719

[43] Pryce J, Richardson M, Lengeler C. Insecticide-treated nets for preventing malaria. Cochrane Database Syst Rev. 2018;11:CD000363. Epub 2018/11/07. doi: 10.1002/14651858.CD000363.pub3. ; PubMed Central PMCID: PMC6418392. 30398672 PMC6418392

[44] World Health Organization. Control and elimination of *Plasmodium vivax* malaria: a technical brief: World Health Organization; 2015.

[45] Kearney EA, Agius PA, Chaumeau V, Cutts JC, Simpson JA, Fowkes FJ. *Anopheles* salivary antigens as serological biomarkers of vector exposure and malaria transmission: A systematic review with multilevel modelling. medRxiv. 2021:2021.09.14.21263589. doi: 10.1101/2021.09.14.21263589 PMC8860437 34939933

[46] Ya-Umphan P, Cerqueira D, Parker DM, Cottrell G, Poinsignon A, Remoue F, et al. Use of an Anopheles Salivary Biomarker to Assess Malaria Transmission Risk Along the Thailand-Myanmar Border. J Infect Dis. 2017;215(3):396–404. Epub 2016/12/10. doi: 10.1093/infdis/jiw543. ; PubMed Central PMCID: PMC5853934. 27932615 PMC5853934

[47] Brady OJ, Godfray HC, Tatem AJ, Gething PW, Cohen JM, McKenzie FE, et al. Vectorial capacity and vector control: reconsidering sensitivity to parameters for malaria elimination. Trans R Soc Trop Med Hyg. 2016;110(2):107–17. Epub 2016/01/30. doi: 10.1093/trstmh/trv113. ; PubMed Central PMCID: PMC4731004. 26822603 PMC4731004

