# Supplementary material for: Outdoor residual spraying for malaria vector-control in Kayin (Karen) state, Myanmar: A cluster randomized controlled trial
Source: PLoS One. 2022 Sep 9;17(9):e0274320. doi: 10.1371/journal.pone.0274320 (PMC9462579; doi:10.1371/journal.pone.0274320)
Supplement: S1 Table — (DOCX) [file pone.0274320.s001.docx]

### S1 Table. Randomization table used to allocate intervention to the villages enrolled in the study.

| **Village** | **Proportion of *Anopheles* among the total number of collected mosquitoes** | | | **Mean human-biting rate (no. bites /person /night)** | | | **Mean HBR / Mean CBR ratio** | | | | **Score** | **Block** | **Group** |
| --- | --- | --- | --- | --- | --- | --- | --- | --- | --- | --- | --- | --- | --- |
|  | **n/N** | **estimate** | **rank** | **n/N** | **estimate** | **rank** | | **n/N** | **estimate** | **rank** |  |  |  |
| MP-0586 | 497/1285 | 0.39 | 10 | 120/100 | 1.2 | 12 | | (120/100) / (377/10) | 0.032 | 6 | 142 | 1 | control |
| HP-0574 | 2114/9695 | 0.22 | 12 | 677/100 | 6.77 | 6 | | (677/100) / (1437/10) | 0.047 | 3 | 141 | 1 | ORS |
| TW-0568 | 2479/8203 | 0.3 | 11 | 678/100 | 6.78 | 5 | | (678/100) / (1801/10) | 0.038 | 5 | 130 | 2 | control |
| TK-0814 | 1034/2544 | 0.41 | 9 | 354/100 | 3.54 | 9 | | (354/100) / (680/10) | 0.052 | 1 | 118 | 2 | ORS |
| DL-0583 | 838/1745 | 0.48 | 7 | 270/100 | 2.7 | 11 | | (270/100) / (568/10) | 0.048 | 2 | 105 | 3 | ORS |
| PW-0563 | 3670/7945 | 0.46 | 8 | 646/90 | 7.18 | 4 | | (646/90) / (3024/9) | 0.021 | 9 | 101 | 3 | control |
| HT-0621 | 2502/4280 | 0.58 | 6 | 383/100 | 3.83 | 8 | | (383/100) / (2119/10) | 0.018 | 10 | 94 | 4 | ORS |
| KK-0610 | 6014/10134 | 0.59 | 5 | 904/100 | 9.04 | 3 | | (904/100) / (5110/10) | 0.018 | 11 | 70 | 4 | control |
| TW-0794 | 1389/1987 | 0.7 | 3 | 309/100 | 3.09 | 10 | | (309/100) / (1080/10) | 0.029 | 8 | 68 | 5 | ORS |
| WK-0567 | 4386/6379 | 0.69 | 4 | 1018/100 | 10.18 | 2 | | (1018/100) / (3368/10) | 0.03 | 7 | 53 | 5 | control |
| TM-0798 | 2740/3372 | 0.81 | 1 | 338/50 | 6.76 | 7 | | (338/50) / (2402/5) | 0.014 | 12 | 43 | 6 | ORS |
| TN-0579 | 4969/6435 | 0.77 | 2 | 1462/100 | 14.62 | 1 | | (1462/100) / (3507/10) | 0.042 | 4 | 27 | 6 | control |
