# Supplementary material for: Outdoor residual spraying for malaria vector-control in Kayin (Karen) state, Myanmar: A cluster randomized controlled trial
Source: PLoS One. 2022 Sep 9;17(9):e0274320. doi: 10.1371/journal.pone.0274320 (PMC9462579; doi:10.1371/journal.pone.0274320)
Supplement: S2 Table — (DOCX) [file pone.0274320.s002.docx]

### S2 Table. Insecticide resistance patterns in malaria mosquitoes during baseline surveys.

| **Insecticide / Taxa** | **Proportion of resistant phenotypes** | | | **Proportion of knocked-down mosquitoes** | | |
| --- | --- | --- | --- | --- | --- | --- |
|  | **n/N** | **Mean (%)** | **95% CI** | **n/N** | **Mean (%)** | **95% CI** |
| Lambda-cyhalothrin |  |  |  |  |  |  |
| *An. annularis* | 369/1476 | 25 | 23 - 27 | 1473/1476 | 100 | 99 - 100 |
| *An. barbirostris* | 28/73 | 38 | 27 - 50 | 61/73 | 84 | 73 - 91 |
| *An. culicifacies* | 31/53 | 58 | 44 - 72 | 26/53 | 49 | 35 - 63 |
| *An. dirus* | 0/3 | 0 | 0 - 71 | 3/3 | 100 | 29 - 100 |
| *An. hyrcanus* | 127/173 | 73 | 66 - 80 | 41/173 | 24 | 18 - 31 |
| *An. jamesii* | 159/700 | 23 | 20 - 26 | 698/700 | 100 | 99 - 100 |
| *An. jeyporiensis* | 0/2 | 0 | 0 - 84 | 1/2 | 50 | 1 - 99 |
| *An. karwari* | 1/24 | 4 | 0 - 21 | 24/24 | 100 | 86 - 100 |
| *An. kochi* | 2/55 | 4 | 0 - 13 | 55/55 | 100 | 94 - 100 |
| *An. maculatus* | 117/826 | 14 | 12 - 17 | 823/826 | 100 | 99 - 100 |
| *An. minimus* | 51/431 | 12 | 9 - 15 | 428/431 | 99 | 98 - 100 |
| *An. tessellatus* | 7/47 | 15 | 6 - 28 | 39/47 | 83 | 69 - 92 |
| *An. vagus* | 90/162 | 56 | 48 - 63 | 93/162 | 57 | 49 - 65 |
| Deltamethrin |  |  |  |  |  |  |
| *An. annularis* | 147/836 | 18 | 15 - 20 | 833/836 | 100 | 99 - 100 |
| *An. jamesii* | 9/121 | 7 | 3 - 14 | 121/121 | 100 | 97 - 100 |
| *An. maculatus* | 22/220 | 10 | 6 - 15 | 219/220 | 100 | 97 - 100 |
| *An. minimus* | 1/26 | 4 | 0 - 20 | 26/26 | 100 | 87 - 100 |
| Permethrin |  |  |  |  |  |  |
| *An. annularis* | 27/603 | 4 | 3 - 6 | 603/603 | 100 | 99 - 100 |
| *An. jamesii* | 0/101 | 0 | 0 - 4 | 101/101 | 100 | 96 - 100 |
| *An. maculatus* | 6/147 | 4 | 2 - 9 | 143/147 | 97 | 93 - 99 |
| *An. minimus* | 3/23 | 13 | 3 - 34 | 23/23 | 100 | 85 - 100 |
| Bendiocarb |  |  |  |  |  |  |
| *An. annularis* | 0/473 | 0 | 0 - 1 | 473/473 | 100 | 99 - 100 |
| *An. maculatus* | 1/25 | 4 | 0 - 20 | 25/25 | 100 | 86 - 100 |
| Propoxur |  |  |  |  |  |  |
| *An. annularis* | 5/470 | 1 | 0 - 2 | 468/470 | 100 | 98 - 100 |
| *An. maculatus* | 2/120 | 2 | 0 - 6 | 120/120 | 100 | 97 - 100 |
| DDT |  |  |  |  |  |  |
| *An. annularis* | 17/408 | 4 | 2 - 7 | 395/408 | 97 | 95 - 98 |
